# IgG4-Related Disease Strikes the Cervical Spine: First Description of a Rare Cause for C1 Destruction and Tetraparetic Stenosis

**DOI:** 10.3390/reports9020097

**Published:** 2026-03-26

**Authors:** Joe Mehanna, Steffen-Heinrich Schulz, Sascha Gravius, Christine Schülin, Franz-Joseph Dally, Frederic Bludau

**Affiliations:** 1 Orthopedic and Trauma Surgery Center, University Hospital Mannheim, 68167 Mannheim, Germanyfranz.dally@umm.de (F.-J.D.); frederic.bludau@umm.de (F.B.); 2 Neurological Rehabilitation Center, Schmieder Clinics Heidelberg, 69117 Heidelberg, Germany

**Keywords:** IgG4-related disease, cervical spine, C1 osteolysis, craniovertebral junction, atlantoaxial instability, tetraparesis

## Abstract

**Background and Clinical Significance**: Immunoglobulin G4-related disease (IgG4-RD) is a systemic immune-mediated fibroinflammatory disorder that can mimic infection or malignancy. Spinal involvement is exceedingly rare and usually limited to pachymeningitis or epidural pseudotumors. True vertebral bone destruction has been reported only sporadically. **Case Presentation**: A 54-year-old man presented to our emergency department with severe neck pain after a fall. CT and MRI revealed extensive osteolysis of the C1 posterior arch and odontoid process with atlantoaxial subluxation. Following a second inpatient fall, he developed acute tetraparesis. Emergency posterior occipitocervical fusion (C0–C4) with C1–C2 laminectomy and foramen magnum decompression was performed. Histopathology demonstrated dense lymphoplasmacytic infiltration and fibrosis with up to 36 IgG4^+^ plasma cells per high-power field and an IgG4^+^/IgG ratio > 40%, confirming IgG4-RD. The patient recovered substantial motor function postoperatively and regained independent ambulation after neurological rehabilitation. **Conclusions**: IgG4-RD can rarely present as destructive craniovertebral osteolysis with neurological compromise. Unexplained C1–C2 osteolytic lesions should prompt evaluation for IgG4-RD, a rare but treatable cause of cervical instability.

## 1. Introduction and Clinical Significance

Immunoglobulin G4-related disease (IgG4-RD) is a systemic, immune-mediated condition characterized by dense fibroinflammatory infiltration rich in IgG4-positive plasma cells, potentially involving almost every organ system. Its diagnosis remains challenging due to the wide spectrum of clinical presentations, the absence of pathognomonic laboratory tests, and significant overlap with the inflammatory, infectious, or neoplastic disorders [1,2]. While the pancreas, lacrimal, and salivary glands are most frequently involved, spinal manifestations are exceedingly uncommon [3].

The prevalence of IgG4-RD is not well established. It predominantly affects men, with the average age at presentation in the fifth to sixth decade [4].

The diagnosis of IgG4-RD is clinicopathological, as serum IgG4 elevation alone is neither sensitive nor specific. It requires concordant clinical, radiological, and histopathological features within an appropriate context. The 2019 ACR/EULAR classification criteria define typical organ involvement and exclusion parameters to improve precision [5].

Spinal manifestation of IgG4-RD remains rare and largely limited to hypertrophic pachymeningitis and epidural pseudotumors causing compressive myelopathy [6,7]. True osseous involvement of vertebral structures has been reported only sporadically. Kim et al. described a case of thoracic vertebral destruction (T11) confirmed as IgG4-related spondylitis after En-Bloc spondylectomy, representing one of the first verified examples of spinal bone involvement [3]. Similarly, Park et al. reported osteolytic lesions at the craniovertebral junction with no significant spinal cord compression or tetraparesis, involving the occipital condyle and C1 lateral mass, which required occipitocervical fusion and histopathological confirmation of IgG4-RD [8].

We report the first histopathologically confirmed case of IgG4-related disease involving the C1 vertebra, characterized by osteolytic destruction, C1–C2 subluxation, and severe tetraparetic cervical stenosis. The patient underwent successful posterior occipitocervical fusion (C0–C4) with C1–C2 laminectomy and foramen magnum decompression. This case broadens the recognized clinical, radiological, and surgical spectrum of spinal IgG4-RD and emphasizes the need to consider this entity in the differential diagnosis of destructive craniovertebral junction lesions.

## 2. Case Presentation

A 54-year-old man was brought to the emergency department after several falls at home, presenting with severe cervical pain. His medical history included alcohol dependence, complete tooth loss due to severe periodontitis several years prior, chronic sinusitis with nasal obstruction, and nasal speech secondary to palatal insufficiency. He was taking opioid analgesics for cervical pain. On admission, neurological examination was normal, and no focal deficits were identified.

**Figure 1:**
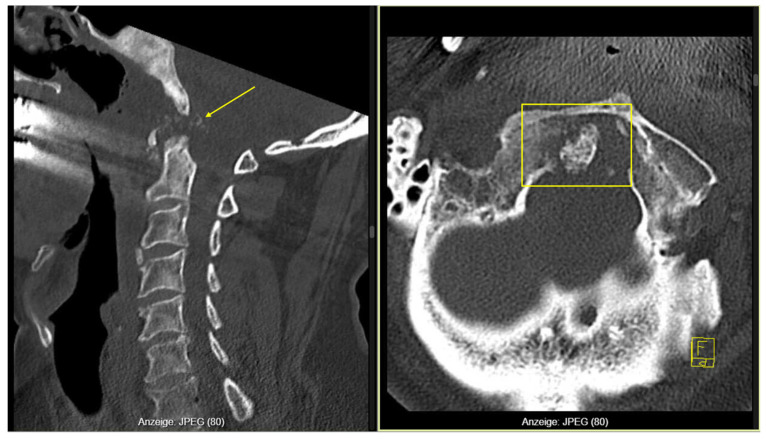
Cervical CT showing C1 osteolysis with Pannus (arrow) and atlantoaxial subluxation (frame).

A cervical CT scan revealed pannus-like soft-tissue proliferation with extensive osteolytic destruction of the right posterior arch of C1, the odontoid process, and the left occipital condyle, associated with atlantoaxial subluxation and rightward displacement of the dens (Figure 1). Due to suspected instability, a rigid cervical orthosis (Miami-J; Össur Americas Inc., Irvine, CA, USA) was applied. Laboratory results showed leukocytosis (16 × 10^9^/L) and CRP 90 mg/L, but blood and urine cultures as well as chest imaging were negative. The patient remained afebrile throughout.

**Figure 2:**
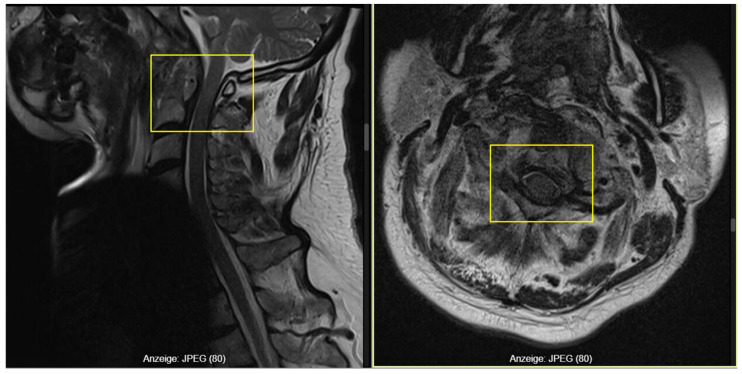
MRI-T2 demonstrating retro-odontoid enhancing lesion (left frame) with a moderate canal stenosis and a preserved CSF rim (right frame).

MRI of the cervical spine demonstrated a contrast-enhancing retro-odontoid lesion (2.6 cm × 0.7 cm) causing moderate canal stenosis with a preserved CSF rim, and additional enhancement in the anterior C2 arch and odontoid process, compatible with inflammatory or infectious atlantoaxial pathology (Figure 2).

**Figure 3:**
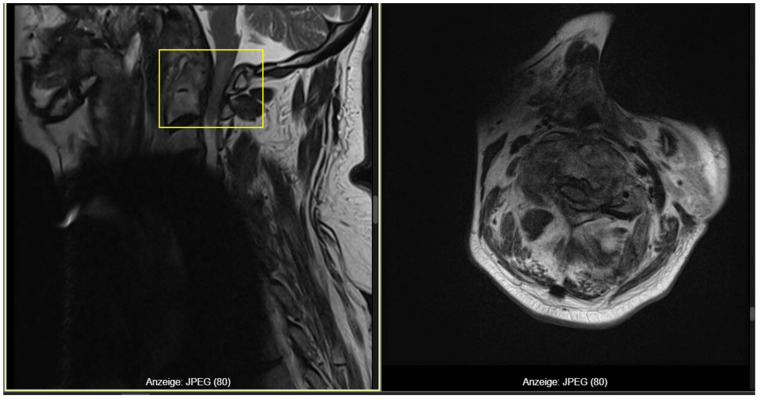
Follow-up MRI-T2 revealing C1–C2 stenosis with myelopathy (frame).

During hospitalization, before the planned surgical intervention, the patient sustained another fall, after which he developed progressive neurological deterioration with fluctuating tetraparesis (2/5–4/5), predominantly on the right side. A repeat MRI revealed progressive C1–C2 canal stenosis, loss of the CSF space, and a faint myelopathic signal at the cervicomedullary junction, consistent with impending high cervical spinal cord compression (Figure 3). Emergency surgery was therefore indicated.

### 2.1. Surgical Technique

Under general anesthesia, the patient was positioned prone in a Mayfield carbon head clamp. After sterile preparation, a midline posterior approach from the occiput to C6 was performed. The nuchal ligament was divided, and the posterior elements of C1–C4 were exposed. The occipital bone was prepared and fixed with a midline occipital plate approximately 2 cm above the foramen magnum. Lateral mass screws were inserted bilaterally at C3–C4, and pars/isthmic screws at C2 based on preoperative CT planning.

**Figure 4:**
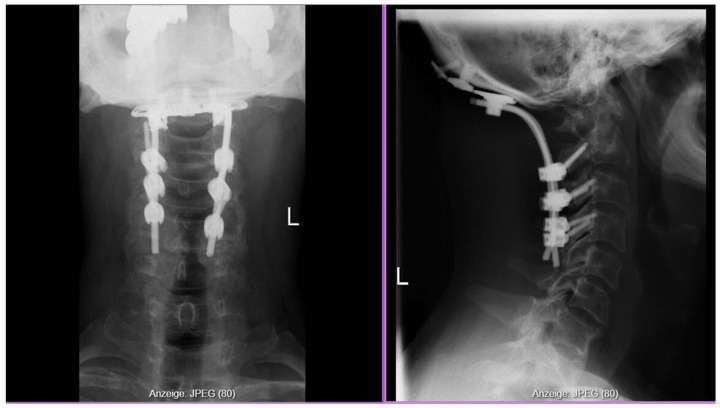
Postoperative X-ray after occipitocervical fusion (C0–C4).

The posterior arch of C1, already pathologically fractured and softened by inflammatory erosion, was removed using a high-speed drill and Kerrison rongeurs. The foramen magnum decompression was then extended cranially, with the posterior rim identified by palpation using a blunt hook to ensure adequate decompression without over-resection. C2 laminectomy followed, including resection of the ligamentum flavum from C0 to C2 for decompression of the foramen magnum and upper cervical canal. A granulation tissue mass (pannus) extending from the ventral odontoid region dorsally was carefully dissected off the dura and excised for histopathological and microbiological analysis. After thorough irrigation and hemostasis, occipitocervical fusion (C0–C4) was completed, consisting of the occipital plate, C2 isthmus screws and C3/4 Massa laterals screws and contoured rods (Figure 4) along with autologous bone graft placed posterolateral to promote fusion.

No dural tears or CSF leakage occurred. Multiple tissue samples and the bony posterior C1 ring containing fibrotic tissue were sent for microbiological and histopathological examination. Intraoperative neuromonitoring (MEPs/SEPs) was not employed due to the emergency setting and the inability to obtain reliable baseline signals, given the patient’s pre-existing fluctuating tetraparesis.

### 2.2. Microbiological Findings

Microbiological findings showed a positive germ detection in one sample only identified as *Staphylococcus aureus*. This finding led to the working diagnosis of a retropharyngeal abscess. Treatment with a fitting antibiotic agent was started.

### 2.3. Histopathological Findings

Histopathological and immunohistochemical analysis of the excised tissue demonstrated dense lymphoplasmacytic infiltration with areas of storiform fibrosis. Immunostaining revealed a marked increase in IgG4-positive plasma cells, with focal counts of up to 36 IgG4^+^ cells per high-power field (HPF) and an IgG4^+^/IgG plasma cell ratio exceeding 40%. The plasma cells were CD138-positive, and kappa/lambda staining confirmed a polyclonal population.

These findings were diagnostic of IgG4-related inflammatory disease involving the C1 vertebra.

### 2.4. Postoperative Course

The patient was transferred to the intensive care unit for neurological and hemodynamic monitoring. Empiric antibiotic therapy with ceftriaxone, flucloxacillin, and rifampicin was initiated.

Neurological function improved immediately postoperatively, with progressive recovery of limb strength. Postoperative CT confirmed correct implant positioning. *Staphylococcus aureus* was isolated from a single intraoperative specimen, and antibiotics were continued for six weeks, followed by oral amoxicillin–clavulanate.

At discharge, the patient had 3+–4/5 strength in all extremities, was able to sit independently and ambulate with a walker, and showed marked improvement compared to his preoperative tetraparesis. The patient was discharged into a special neurologic rehabilitation center. After completing inpatient rehabilitation, he regained independent ambulation and returned to normal daily activities.

## 3. Discussion

The slow onset of tetraplegia due to destructive C1 osteolysis in a patient ultimately diagnosed with IgG4-related disease (IgG4-RD) is a clinical scenario that challenges conventional diagnostic frameworks and highlights the protean nature of this fibroinflammatory disorder. While IgG4-RD is increasingly recognized as a multisystem entity, spinal involvement—particularly with frank bone destruction and catastrophic neurological sequelae—remains exceedingly rare. This case is notable as the first documented instance of IgG4-RD presenting with C1 osteolysis and tetraplegia, expanding the known spectrum of IgG4-RD manifestations and underscoring the need for heightened clinical suspicion in atypical spinal presentations [8,9,10].

Most reported cases of IgG4-RD with spinal involvement manifest as hypertrophic pachymeningitis, epidural or intradural masses, or sclerosing lesions, typically leading to progressive myelopathy or radiculopathy [10,11]. Park et al. described IgG4-RD at the craniovertebral junction with lytic destruction of C1 and the occipital condyle, but without tetraplegia; other reports detail spinal cord compression due to pachymeningitis or pseudotumor formation, but none combine C1 osteolysis, severe neurological deficit, and operative stabilization as seen here [8]. This case thus represents a unique and previously unreported constellation of findings.

The differential diagnosis of destructive craniovertebral junction lesions encompasses pathologies that can closely mimic IgG4-RD. Infectious spondylodiscitis, particularly with *Staphylococcus aureus*, typically presents with disc space involvement and paravertebral abscesses, though the positive culture in our case initially suggested this diagnosis [12,13]. However, IgG4-RD characteristically demonstrates dense fibrotic tissue with storiform pattern rather than purulent collections. Metastatic disease and primary bone tumors (including chordoma and plasmacytoma) represent critical considerations but typically show more aggressive destruction on imaging and malignant cells on histopathology rather than lymphoplasmacytic infiltrates. Rheumatoid arthritis can cause atlantoaxial instability through pannus formation but it occurs in patients with established seropositive disease and lacks the characteristic IgG4^+^ plasma cell infiltration. Other inflammatory conditions, including granulomatosis with polyangiitis and sarcoidosis may present similarly but are distinguished by their distinct histopathological features and serological profiles. The key to diagnosis lies in histopathological confirmation showing >40% IgG4^+^/IgG ratio and characteristic storiform fibrosis, which definitively distinguishes IgG4-RD from its mimics [11,12].

Pathophysiologically, IgG4-RD is characterized by lymphoplasmacytic infiltrates, storiform fibrosis, and obliterative phlebitis. While bone involvement is rare, chronic inflammation can drive local osteoclast activation and pannus formation, leading to destructive lesions and instability. The mechanisms parallel those seen in hypertrophic pachymeningitis, where fibroinflammatory changes extend to adjacent osseous structures, resulting in mass effect and cord compression. The destructive process at C1 in this case is pathophysiologically plausible but exceptionally rare [14,15].

The clinical presentation of IgG4-RD in the spine is often insidious, with progressive pain, neurological deficits, and sometimes autonomic dysfunction. Imaging may reveal mass-like lesions, bone destruction, or diffuse thickening, but these findings are not pathognomonic and can mimic neoplastic, infectious, or other inflammatory conditions [16]. Laboratory findings may include elevated serum or CSF IgG4, but these are neither sensitive nor specific, and histopathology remains the gold standard for diagnosis [11,12,17].

The diagnosis of IgG4-RD in this case was particularly challenging, as the patient was initially referred with a presumed diagnosis of bacterial spondylodiscitis based on a single positive intraoperative culture. However, several factors prompted reconsideration: the questionable infectious etiology with only one positive specimen out of four, the fibrotic tissue character, and the slow clinical onset. A thorough review of the patient’s history revealed seemingly unrelated findings—complete tooth loss due to severe periodontitis, chronic sinusitis with nasal obstruction, and palatal insufficiency causing nasal speech—that collectively raised suspicion for a systemic fibroinflammatory condition. Initial differential considerations included ANCA-associated vasculitis and IgG4-RD. This clinical reasoning, integrating apparently minor historical details, led to targeted histopathological re-evaluation with IgG4 immunostaining, ultimately confirming the diagnosis. A remote history of possible idiopathic pancreatitis was also noted, though not confirmed. This case illustrates that when conventional diagnoses remain uncertain, associating seemingly unrelated clinical findings may reveal the underlying etiology. The patient was referred for rheumatologic evaluation after discharge for long-term immunosuppressive management.

Management of IgG4-RD with spinal involvement requires early surgical decompression and stabilization in the setting of neurological compromise, combined with immunosuppressive therapy. Glucocorticoids are the first-line treatment, with rituximab reserved for refractory or relapsing disease, as recommended by international consensus [18]. Multidisciplinary care is essential, and favorable functional recovery is possible even after severe local destruction, provided timely intervention is achieved [8,10,15,16,18]. Maintenance therapy and long-term follow-up are recommended to monitor for recurrence and manage chronic disease activity [18].

Early recognition and combined surgical and immunosuppressive therapy are critical for optimal outcomes. Clinicians should maintain a high index of suspicion for IgG4-RD in atypical spinal presentations, especially when conventional causes are excluded [8,9,11,18]. The evolving understanding of IgG4-RD pathogenesis, diagnostic criteria, and therapeutic options underscores the need for ongoing research and multidisciplinary collaboration in the management of this complex disease [19]. Gathering a thorough patient history (idiopathic pancreatitis for instance) is essential in maintaining suspicion for rare conditions.

Diagnosis of the IgG4-RD in this case was especially challenging due to the germ detection mimicking for an infectious case of spinal stenosis.

After the diagnosis the patient was presented to the rheumatology department and managed there. Systemic staging was ongoing and decisions regarding immunosuppressive therapy were pending.

## 4. Conclusions

IgG4-related disease, though rare, can involve the cervical spine and mimic infectious or neoplastic pathology, leading to destructive instability and neurological compromise. Early recognition is essential, as timely surgical stabilization and appropriate immunosuppressive therapy can achieve excellent recovery. Unexplained osteolytic lesions at the craniovertebral junction should prompt thorough evaluation for IgG4-related disease as a potential, yet treatable, underlying cause.

## Data Availability

The original data presented in the study are included in the article, further inquiries can be directed to the corresponding author.
